# Editorial: World AIDS Day 2022: putting ourselves to the test: achieving equity to end HIV

**DOI:** 10.3389/fpubh.2024.1478645

**Published:** 2024-09-13

**Authors:** Diego Ripamonti, Segundo R. Leon

**Affiliations:** ^1^Papa Giovanni XXIII Hospital, Bergamo, Italy; ^2^School of Medical Technology, San Juan Bautista Private University, Lima, Peru

**Keywords:** HIV, HAART, health equity, disparity, PrEP

Outstanding results have been achieved over the last four decades in terms of antiretroviral (ARVs) access and coverage (1) thanks to the introduction of generic medications worldwide (2) that have helped many regions to achieve the UNAIDS target (3, 4) of 95-95-95% of diagnosed, treated and virologically suppressed People Living With HIV (PLWH). Now, an additional target, defined as the 4th 90, has been added to go beyond the simple virological control and aim at healthy aging in such a diverse population (5).

The WHO (6), which has been recommending the “treatment for all” approach since 2015, has also focused on universal and equitable access in order to optimize the provision and uptake of ARVs in all countries and, in particular, among disproportionately affected communities. For instance, despite more than 30 years of massive mobilization by national governments and external donors, in Sub-Saharan Africa (SSA, the most affected region by the HIV epidemic), equity in HIV programs remains a challenge (7). Equity in HIV management means providing similar opportunities to minorities, irrespective of age or gender, and, most importantly, across different countries, recognizing that vulnerable populations are those most in need and least supported (8).

Treatment equity is a challenge in terms of testing rates, ART coverage, adherence to visits and medication, and virological suppression rates. This happens because of educational, structural, social, cultural, political and economic reasons (9, 10) and challenges in implementation vary by region, country, and community, making equitable opportunities a mirage. Of course, roadblocks are present in all countries, although at different levels and magnitudes and for different reasons, requiring distinct regionally-based approaches. This implies a deep understanding of the geographical diversities in terms of health system capacity, economic scenarios, key populations, political settings, religious beliefs and regional stigma, without forgetting the needs and preferences of PLWH. Bridging the gap is mandatory as high levels of ARV coverage and prevention strategies are needed in order to achieve HIV infection control and, possibly, elimination.

The aim of this Research Topic is to focus on the persistent gap in equitable access to HIV care around the world. The present Research Topic sheds light on local experiences from Africa to Qatar, Iran, India, Taiwan, China, Australia and North America, and across different settings and key populations, addressing persistent cultural barriers, unmet needs and delays of health systems.

## HIV testing

HIV testing is the most cost-effective measure for the prevention and control of HIV transmission, and despite significant increases in access to HIV care over the past two decades, disparities are still important (8). In Sub-Saharan Africa there is still wide variation in the frequency of testing with clear geographical hotspots of inequality (11).

Alie and Negesse used a machine learning approach in an attempt to predict which adolescents are at a high risk of infection in Ethiopia. Based on data from 4,502 adolescent respondents, they identified predictors for targeted screening strategies in this setting.

Zhang et al. conducted a qualitative study of 26 Asian-born MSM living in Australia about their attitude toward HIV self-testing as an additional testing opportunity. This option may be of help in reducing the risk of transmission in high-risk populations.

Farag et al. reported on HIV testing in Qatar, a country where ~90% of the total population comprises expatriates and has a single provider of outpatient HIV services. HIV testing is extensively conducted as part of many clinical situations or common practices (such as applying for a job or university, before marriage, or before moving to Qatar), although no data are available on the sexual behavior of both residents and migrants.

## Stigma and vulnerability

The stigma of HIV infection has been a major barrier to voluntary and confidential HIV testing. In fact, more than 40 years into the global HIV pandemic, social and internalized stigma remains one of the major determinants of low levels of testing, fear of disclosure, isolation and depression (12).

Bouabida et al. addressed the obstacles to individual engagement in the HIV care cascade. These range from stigma and vulnerability to mental health problems and payment models, confirming that treatment adherence is only one part of HIV care.

Ferri France et al. reported on a peer-led HIV self-stigma intervention aimed at improving the self-worth and well-being of 62 adolescents and young PLWH in Zimbabwe. This is a qualitative study of the perceived impact of a self-stigma intervention, which has rarely been explored in young individuals.

Goodman et al. reported on how HIV stigma still predicts subsequent reporting of ever having been tested; the authors have promoted an experimental approach to building a generative context for community-led HIV prevention, now involving over 10,000 rural Kenyans in 39 villages. It is a creative approach aimed at overcoming the persistent barrier to HIV control in rural areas.

Key populations still face widespread criminalization. For instance, a negative HIV test is required for migrant workers who want to stay in a country such as Russia (13).

Skuban-Eiseler et al. reported on 28 PLWH who experienced restrictions regarding their access to healthcare, mostly in Russia and Ukraine, and mostly detainees, creating important barriers in the fight against HIV. Decriminalization should be the very first step toward acceptance, inclusion and openness to testing.

There is still a long way to go before stigma and discrimination can be overcome worldwide.

## Prevention strategies

### Pre-exposure prophylaxis

Oral (14–16) and long-acting (LA) PrEP options (17, 18) have been shown to be very effective in reducing the risk of HIV acquisition, overcoming the limitations of adherence and lack of persistence associated with oral regimens, and are considered the most effective and potentially game-changing opportunities in the fight against HIV to date (18, 19). However, PrEP implementation is slowed down by social and structural barriers for some groups of individuals, making them vulnerable people (19, 20). Oral PrEP programs among adolescent women in Africa have shown a high discontinuation rate (up to 80%) at 6 months (21). Black/African American and Hispanic/Latino individuals, who account for 47 and 39% of estimated new infections in the US (22) have a low level of PrEP uptake (19). In Europe, 13 countries reported that PrEP had not even been implemented by 2023 (23) confirming the slowness of the process. Adolescent girls, who account for 76% of all new infections worldwide (10–19 years) (24), need strong PrEP interventions.

Ung et al. explored the preferences for HIV prevention strategies (PrEP, condom use or no strategy) among 286 Asian-born MSM living in Australia. The higher preference for the PrEP option (52%) confirms its high attractiveness. PrEP needs to be implemented rapidly and extensively in order to cover all the geographical areas, diverse populations, and especially the most vulnerable.

### Premarital and antenatal testing

Antenatal testing is a crucial step in preventing mother-to-child transmission of HIV. Inequalities in the use of health services in pregnant women in Sub-Saharan Africa have been confirmed (25), with significant differences between countries, as only 74% of women were tested for HIV during antenatal care. Socioeconomic status, area of residence and level of education/knowledge about HIV/AIDS influenced testing rates.

Birhanu et al., who interviewed more than 10,000 women in Ethiopia, confirmed that only 21% of study participants reported pre-marital testing which is quite alarming as HIV testing should be part of a public health program.

Tsega et al. report on the low uptake (34%) of HIV testing during prenatal services in 4,152 women in Ethiopia. Level of education, recent health visits, stigma-free attitudes and not living in rural areas are associated with the rate of testing. Such demographic and cultural determinants are not new, unknown or unpredictable, confirming the persistence of traditional barriers.

Zhou et al., in a meta-analysis, reviewed the effectiveness of interventions to improve ART adherence in 2,900 pregnant women, according to nine studies. Their findings acknowledge the role of enhanced standard of care (±supporter) and device reminders as important tools to improve adherence during pregnancy.

## Key populations

As addressed by Bouabida et al., many are the barriers to health system access faced by PLWH, which may vary according to country and setting, in particular for vulnerable populations.

### Minorities

For a number of reasons minorities lack HIV literacy (defined as one's ability to acquire, communicate and manage basic health information and services), are at higher risk of infection, have lower ARV coverage, and are therefore less likely to be on treatment and virologically suppressed (26).

Shi et al. reported on the average viral suppression rate among PLWH in 46 counties in South Carolina (USA). Their findings revealed that counties with high racial/ethnic residential segregation were more likely to have virologically unsuppressed individuals, confirming that health disparities in HIV treatment and care exist in underserved populations.

An et al. investigated factors associated with patient activation (defined as an individual's knowledge, skills and confidence) among Yi minority PLWH in China. Their low level of activation was associated with lower ART efficacy, suggesting the necessity for targeted strategies.

### Men who have Sex with Men

MSM are still at high risk for HIV and other sexually transmitted infections (STIs) worldwide.

Guo et al. reported on the risk of HIV and STIs among 572 MSM using mobile geosocial networking applications in southern China. Although app users were more likely to engage in high-risk sexual behaviors, the prevalence of HIV and other STIs appeared similar to non-app users.

### People who inject drugs

New HIV infections among intravenous drug users (IVDUs) are increasing in some European countries (27). In 2022, no European country had achieved all 95-95-95 targets (28) for the continuum of care among IVDUs, confirming that they are less likely to be diagnosed, linked to care and virologically suppressed.

Roshanfekr et al. reported low levels of testing in IVDUs in Iran, with only 70% of them reporting lifetime HIV testing. These data suggest that much work still needs to be done to reach individuals traditionally at risk.

### Children

In 2021, an estimated 2.73 million children aged 0–19 years were living with HIV, with the majority (80–90%) in sub-Saharan Africa (29). In 2022, only 52% of children aged 14 or younger were on treatment, while 60% of children aged 5–14 years were still not being treated (30). In a meta-analysis including 4,422 young participants, the prevalence of good treatment adherence was 73% (31). Virological response in this subset is still a challenge due to adherence issues, family and patient factors, education, stigma, and relationships with healthcare personnel, and is hampered by limited pediatric formulations (32).

Ally et al. described the determinants of virological suppression in 1,980 children in Tanzania. Despite a remarkable rate of virological suppression (85%), predictors of successful outcomes include the presence of support groups, health insurance, food security, and family size, confirming the multifaceted nature of treatment success.

Lao et al. reported on the 13-year treatment efficacy of an LPV/r-based regimen in 458 children in China. Their findings confirm the lower ART performance in rural settings and the insufficient implementation of INSTI-based regimens in the pediatric population.

Chakakala-Chaziya et al. compared the clinical outcomes in 27,229 PLWH of different age groups in Malawi, suggesting that younger and older adolescents were less likely to achieve virological suppression compared to adults, mainly due to adherence issues.

### Older people

A growing number of people living with HIV are aging, with approximately one-fifth of this population aged 50 years and older worldwide. PLWH experience more comorbidities and geriatric syndromes than aged-matched negative individuals, along with social isolation, stigma, mental health problems and care integration challenges that deserve further study (33).

### Late presenters

Late presentation (i.e., HIV diagnosis with a CD4 count < 350 cells/μL or an AIDS-defining event) is one of the major obstacles to achieving control of the HIV epidemic worldwide. A recent European survey reported that 28,889 PLWH (50.4% of those included between 1981 and 2019) were classified as late presenters (34).

Xu et al. presented real-world data on HIV late presentation and its determinants in China. A high rate (57% out of 2,300 newly diagnosed individuals) were late presenters, confirming the current issue and the need for targeted measures.

Sharafi et al. estimated the duration of delayed diagnosis in Iran through a CD4 depletion model analysis. Many PLWH have no history of regular HIV testing, they are unaware of their disease and delay the opportunity to seek treatment and avoid transmission.

## Quality of life in PLWH

QoL, which has been set up as the 4th 90 in the UNAIDS goals, refers to the ability of PLWH to experience healthy aging, which involves a number of challenges.

Senkoro et al. investigated QoL and its associated factors among 500 PLWH aged >60 in Uganda. Their findings identify both geriatric syndrome and financial stability as factors affecting the QoL and help focus on the peculiar aspects of older PLWH.

Zhong et al. found a relatively low level of QoL in 401 PLWH in China. These findings highlight how comorbidities and mental health issues may affect their personal wellbeing, especially in older adults.

Krulic et al. provided an implementation study of an HIV peer navigation program in Australia. The aim is to describe how the peer navigation relationship can help provide insights and a sense of “acceptance, belonging and reassurance”, favoring connection to services and community while improving the quality of life for PLWH.

Bernard et al. submitted a qualitative study of Group Interpersonal Therapy for the treatment of depression in PLWH in Senegal, focusing on this neglected issue, especially in Sub-Saharan Africa, which requires strong investment and is a crucial point for the QoL of this population.

## HIV-2 infection

Finally, there is a need to fill the gap for HIV-2 infection (less common compared to the HIV-1 epidemic) as limited treatment options are available for this setting due to fewer effective ARV classes, fewer clinical studies of second-line alternatives, a higher risk of resistance at failure and a scarcity of randomized trial data to guide treatment choice (35).

## Closing the gaps in the HIV epidemic

Implementation and access to treatment and prevention tools are still lagging in many countries because of unresolved issues. No single tool or option will fit all countries and settings or may be sufficient to put an end to the HIV epidemic. Cooperation and creative strategies should be tailored, to understanding the diverse and multifaceted scenarios.

Critical steps should be pursued as follows:

- Promote public health campaigns to reduce HIV-associated stigma, including through creative approaches (Goodman et al.) as it still represents a major barrier in the fight against HIV.
- Channel higher efforts into regions with high HIV rates to further expand ART coverage.
- Understand and recognize the disparities in ARV uptake, to identify and reach key populations.
- Find alternative ways to prescribe, deliver and monitor both treatment and prevention tools, prioritize free PrEP options, favor LA strategies and support their implementation, uptake and adherence, and allow individuals to purchase PrEP online (23, 36). PrEP prescription and monitoring can be largely mediated by trained nurses to overcome the shortage of clinicians to whom individuals can be referred at the beginning of the process or as needed.
- Support integration strategies (37).
- Expand treatment opportunities for individual with drug-resistance viruses.
- Promote QoL, including mental health.
- Being creative and innovative in HIV management can help be successful. Technology and telehealth can promote awareness and antiviral uptake, help overcome time and distance barriers to accessing or monitoring services, and improve adherence and retention in care for minorities where PrEP use is a challenge (38, 39).

HIV testing along with stopping transmission through universal treatment and PrEP implementation are the cornerstones of HIV prevention, and all strategies will fail as long as inequalities survive. Disentangling all the critical issues is mandatory to stop the epidemic at a time when HIV eradication is still far from reality and an effective vaccine is not available.
